# Identification of differentially expressed genes-related prognostic risk model for survival prediction in breast carcinoma patients

**DOI:** 10.18632/aging.203178

**Published:** 2021-06-26

**Authors:** Jinyu Li, Gena Huang, Caixia Ren, Ning Wang, Silei Sui, Zuowei Zhao, Man Li

**Affiliations:** 1 Department of Breast Oncology, The Second Hospital of Dalian Medical University, Dalian, Liaoning 116023, China; 2 Department of Respiratory Medicine, The Second Hospital of Dalian Medical University, Dalian, Liaoning 116023, China; 3 Institute for Genome Engineered Animal Models of Human Diseases, Dalian Medical University, Dalian, Liaoning 116044, China; 4 Institute of Cancer Stem Cell, Dalian Medical University, Dalian, Liaoning 116044, China; 5 Department of Breast Surgery, The Second Hospital of Dalian Medical University, Dalian, Liaoning 116023, China

**Keywords:** breast cancer, differentially expressed genes, prognostic risk model, prognostic outcome, Cox regression

## Abstract

Since the imbalance of gene expression has been demonstrated to tightly related to breast cancer (BRCA) genesis and growth, common genes expressed of BRCA were screened to explore the essence in-between. In current work, most common differentially expressed genes (DEGs) in various subtypes of BRCA were identified. Functional enrichment analysis illustrated the driving factor of deactivation of the cell cycle and the oocyte meiosis, which critically triggers the development of BRCA. Herein, we constructed a 12-gene prognostic risk model relative to differential gene expression. Subsequently, the K-M curves, analysis on time-ROC curve and Cox regression were performed to assess this risk model by determining the respective prognostic value, and the prediction performance were ascertained for both training and validation cohorts. In addition, multivariate Cox regression was analysed to reveal the independence between risk score and prognostic stage, and the accuracy and sensitivity of prognosis are particularly improved after clinical indicators are included into the analysis. In summary, this study offers novel insights into the imbalance of gene expression within BRCA, and highlights 12 selected genes associated with patient prognosis. The risk model can help individualize treatment for patients at different risks, and propose precise strategies and treatments for BRCA therapy.

## INTRODUCTION

As the most common malignant tumor in women, breast cancer has exhibited the fifth highest death rate after stomach cancer worldwide [1]. With the progressive increase of both incidence and mortality, it is crucial to evaluate the prognosis in clinical environment and to propose an appropriate therapeutic regimen for malignant tumor patients. In clinical practice, TNM staging is generally used, but it may lead to an entirely distinct prognosis in the same situation [2]. Therefore, a more precise and valuable method is highly desirable to predict outcomes.

The past decade has emerged several novel technologies to explore cancer's molecular characteristics, especially with the rapid advancement of high-throughput next-generation sequencing (NGS), bioinformatics analyses, machine learning, and gene microarray technique. These techniques extremely contributed to early diagnosis of tumors, prognosis prediction, and individualized treatment. In addition, the bioinformatics-based biomarker discovery renders a deeper understanding on disease-related regulatory pathways and molecular mechanisms. To identify high-risk patients, gene risk models are established via bioinformatics analysis using clinical information and gene expression data. More studies have been addressed on establishing gene risk models, resulting from the measuring capability of mRNA expression by NGS and microarray. Particularly, several risk models have played an excellent role in predicting the prognosis outcomes, including autophagy-related gene models, immune cell infiltration-related gene models, nomograms, and so on [3–6]. However, these models elucidated the prognosis of BRCA from the perspective of a single functional genome, which limited their prediction results.

The occurrence and development of tumors are highly relative to the accumulative changes in tumor suppressor genes and oncogenes [7]. The differentially expressed genes (DEGs) play varying roles during different periods of the occurrence and distinct developmental stages of cancer [8]. Genes' abnormal expression has been previously reported to accelerate the progression of malignancies, and DEGs have been targeted as a novel treatment approach in several antitumor clinical types of research [9, 10].

In this study, high-throughput mRNA expression profiles from distinct regions and races have been investigated, focusing on the differences between breast cancer and adjacent mammary tissue hence to identify potential genetic biomarkers. The selected DEGs profiles were incorporated with TCGA-BRCA clinical data to explore DEGs' role in prognosis. Moreover, the independent prediction of BRCA patients' outcomes was achieved using a risk model based on 12 DEGs-related signatures. As such, the risk prediction model is demonstrated as a reliable prognostic marker in BRCA patients. On the other hand, our functional biomarker-based study also provides a novel alternative to predict prognosis in BRCA patients.

## RESULTS

### Identification of DEGs in BRCA

**Figure 1:**
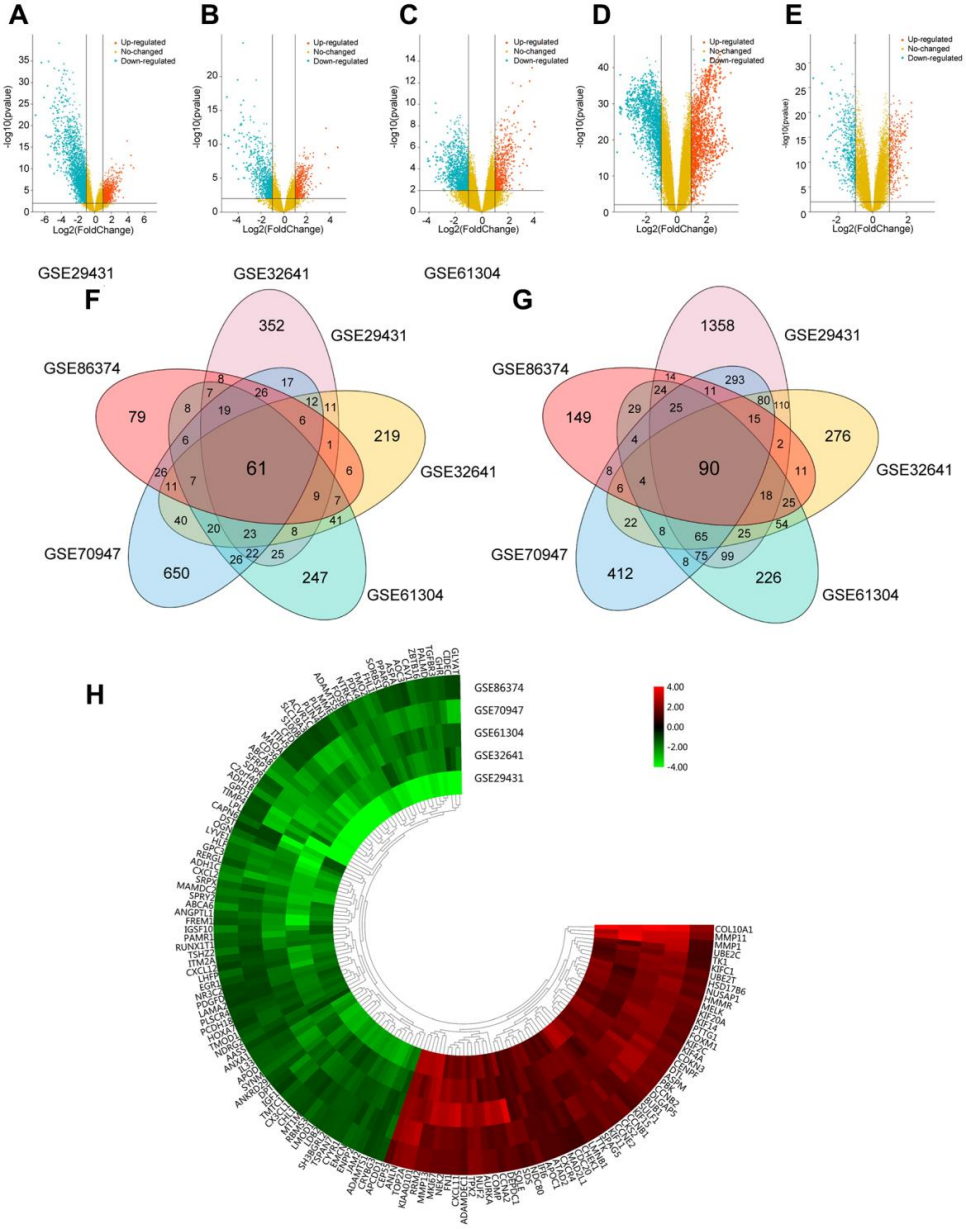
**Foundation of DEGs in five GEO databases.** (**A**–**E**) The display of DEGs in each database by volcano plots. Datasets from GSE29431 (**A**), GSE32641 (**B**), GSE61304 (**C**), GSE70947 (**D**) and GSE86374 (**E**). Orange: Up-regulated genes (logFC ≥ 1.0, adj. *P* < 0.05); Blue: Down-regulated genes (logFC ≤ -1.0, adj. *P* < 0.05); Yellow: Genes with no significance. (**F**–**G**) A Total of 61 significantly upregulated genes (**F**) and 90 significantly downregulated genes (**G**) were screened from the five GEO databases. (**H**) Hierarchical clustering heatmap showed expression of 151 DEGs in five GEO databases. Red: higher expression genes, green: lower expression genes.

To identify DEGs in this study, breast carcinoma samples (479 cases) and normal breast tissues (206 cases) were randomly collected from different regions and races. Using the limma package, we identified 3065 DEGs in GSE29431, 1293 DEGs in GSE32641, 1315 DEGs in GSE61304, 2252 DEGs in GSE70947 and 722 DEGs in GSE86374. DEGs in two representative samples from each of the five expression profiling databases are shown in the volcano plots (Figure 1A–1E). In Figure 1F and 1G, 61 upregulated genes and 90 downregulated genes are in common (|log2Fold Change| >1, adj.*p* < 0.05), which are displayed in the heatmap in five databases (Figure 1H).

### Functional enrichment of DEGs and PPI network construction

**Figure 2:**
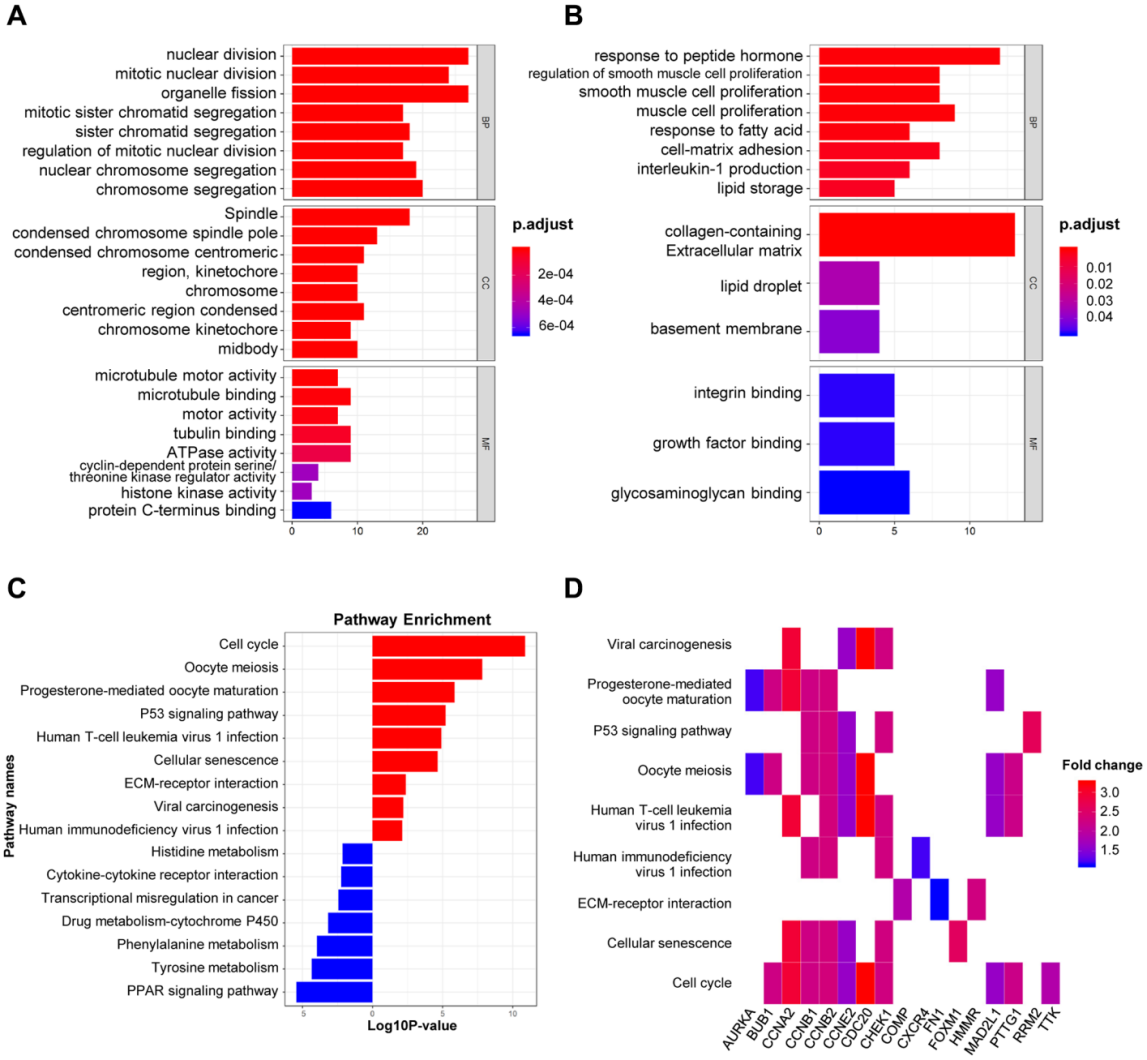
**GO and KEGG analysis of DEGs in BRCA.** (**A**–**B**) The biological processes, cellular components, and molecular functions of 61 up-regulated DEGs (**A**) and 90 down-regulated DEGs (**B**) were displayed by GO analysis. (**C**) The signaling pathways of 151 DEGs were displayed by KEGG analysis. (**D**) Heatmap of the significant enrichment results in the KEGG pathway.

The biological roles of 151 DEGs were further investigated using GO and KEGG pathway analysis. There are three functional categories in GO analysis: 1) in terms of biological process (BP), upregulated DEGs were merely enriched in nuclear division and organelle fission, while downregulated ones were enriched in peptide hormone and smooth muscle cell proliferation; 2) in terms of cellular component (CC), upregulated DEGs were involved in spindle, condensed chromosome and spindle pole, while downregulated ones were involved in collagen-containing extracellular matrix, lipid droplet and basement membrane; 3) in terms of molecular function (MF), microtubule motoring and binding activities were remarkably relative to upregulated DEGs, while downregulated ones were merely involved in integrin and growth factor and glycosaminoglycan binding (Figure 2A–2B). Additionally, KEGG pathway analysis demonstrated the participated roles of most upregulated genes in cell cycle and oocyte meiosis. As a contrast, downregulated genes were merely involved in PPAR signaling pathway and tyrosine metabolism (Figure 2C). The heatmap indicated the relationship between DEGs and enriched KEGG pathways (Figure 2D). Lastly, there are 151 nodes and 1169 edges in the PPI network of DEGs (51 upregulated genes and 90 downregulated genes, *p* = 1.0e-16), and the highest degree is determined at 48 (Supplementary Figure 1).

### Creation and validation of OS-related prognostic risk signature by cox regression

**Figure 3:**
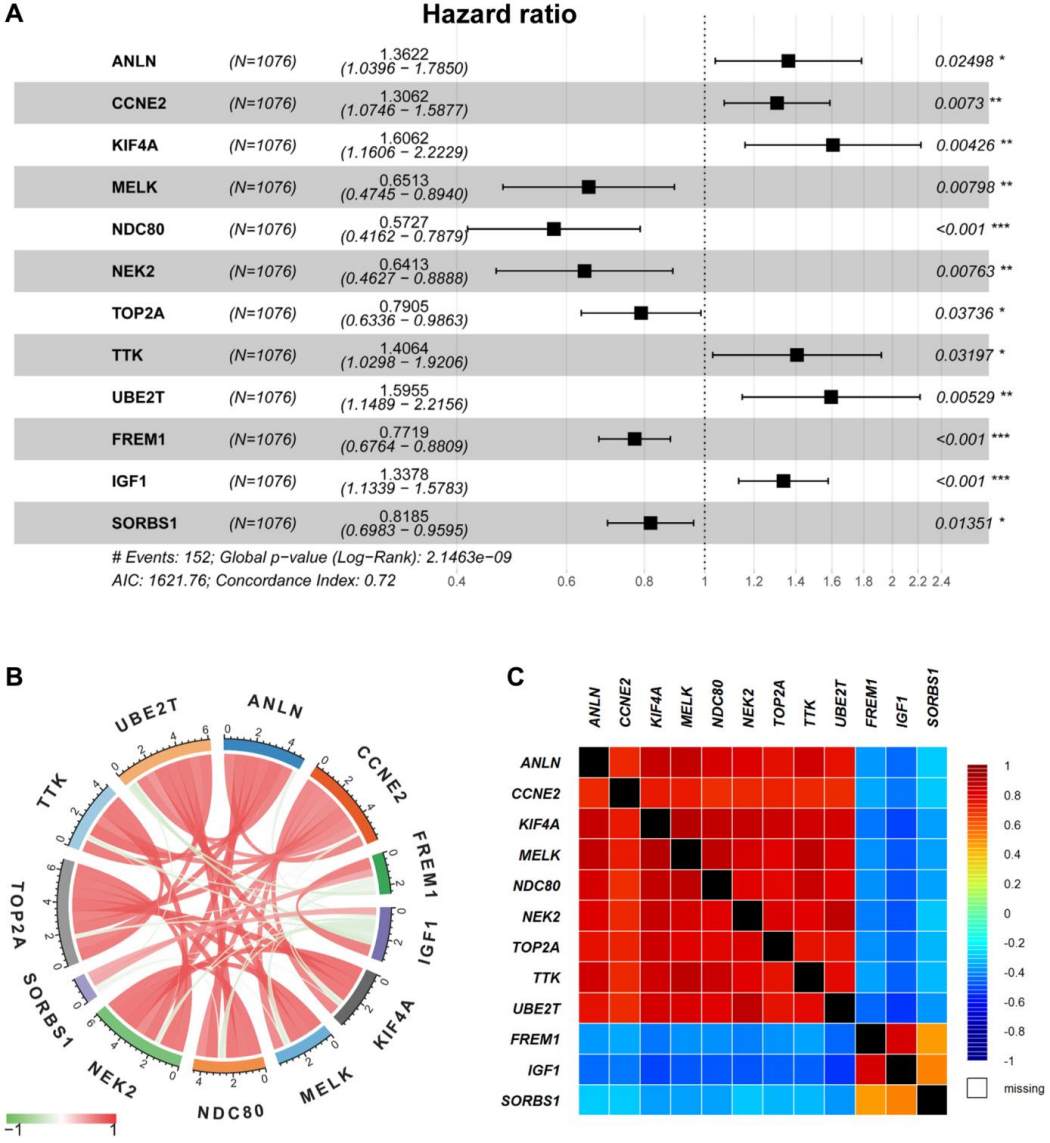
**Multivariable Cox regression and correlation analysis.** (**A**) The multivariable Cox regression analysis of 12 selected DEGs were displayed by forest plot. (**B**–**C**) The correlation analysis of 12 selected DEGs was displayed by Pearson’s correlation (**B**) and the bc-GenExMiner software (**C**), respectively.

TCGA-BRCA and GEO databases were employed for model training (*n* = 1076) and validation (*n* = 408), respectively. As indicated in Supplementary Table 1, there are 31 genes correlated to OS in the TCGA-BRCA cohort upon the univariate Cox regression analysis. Furthermore, the significant OS-related DEGs were identified using multivariate cox regression, in which 12 genes could serve as potential prognostic predictors in BRCA patients (Figure 3A). In Figure 3B and 3C, the correlation analysis suggested that 12 selected genes were cross-interacted.

Besides, genetic alteration of 12 risk-related genes was proceeded to identify their performance in BRCA patients (Supplementary Figure 2). PPI results suggested that most of the 12 selected genes were significantly intercorrelated (*p* < 1.0e-16, Supplementary Figure 3). Subsequently, the survival condition was evaluated in Kaplan Meier-Plotter datasets, indicating that all selected genes were included in the prognostic risk model with a promising survival predicting performance (Supplementary Figure 4).

**Figure 4:**
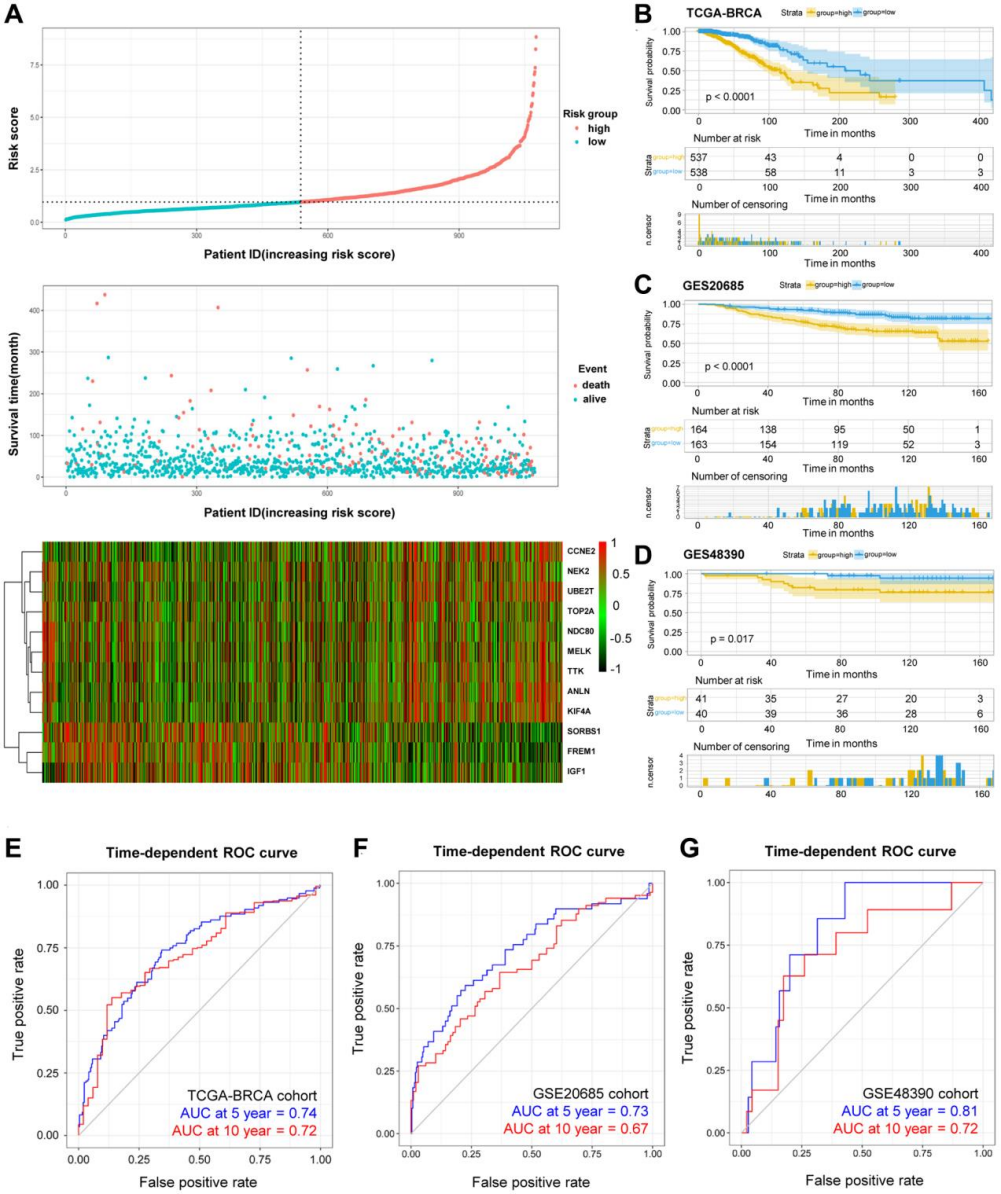
**OS-related prognostic risk model of BRCA patients.** (**A**) The display of prognostic risk model with risk score, patient survival time and status in TCGA-BRCA database. (**B**–**D**) The K-M survival curves of the high- and low-risk group of TCGA-BRCA cohort (**B**) and validation GSE20685 cohort (**C**) and GSE48390 cohort (**D**). (**E**–**G**) The prediction of 5- and 10-year survival in TCGA-BRCA cohort (**E**) and GSE20685 cohort (**F**) and GSE48390 cohort (**G**) by Time-ROC.

Lastly, the formula was established for the prognostic risk model by referring to multiple Cox regression (Risk score = 0.30912 × ANLN expression (Exp-ANLN) + 0.26714 × Exp-CCNE2 + 0.47389 × Exp-KIF4A – 0.42876 × Exp-MELK – 0.55747 × Exp-NDC80 – 0.44425 × Exp-NEK2 – 0.23508 × Exp-TOP2A + 0.34102 × Exp-TTK + 0.46718 × Exp-UBE2T – 0.25889 × Exp-FREM1 + 0.29101 × Exp-IGF1 – 0.20025 × Exp-SORBS1). The respective risk score was determined in the training cohort, patients were then divided into high-risked group or low-risked group (Figure 4A), in accordance to the calculated median risk scores. Using Kaplan-Meier survival analysis, high-risked patients exhibit lower OS rates than that of low-risked patients in the training cohorts (*p* < 0.0001, Figure 4B).

The predictive risk model on prognosis was further investigated, and the established and acquired formula was applied for other 408 BRCA patients in separate cohorts. The validation cohorts consisted of GSE20685 (*n* = 327) and GSE48390 (*n* = 81) databases, including mRNA expression, survival status and survival time. Similarly, patients were group-categorized according to their calculated risk scores. For patients with higher risk score in the validation cohorts (Figure 4C–4D), the worse OS rates were observed using Kaplan-Meier survival analysis. Moreover, receiver operating characteristic (ROC) curves were plotted to investigate the time-dependent dependability of the risk model. As shown in Figure 4E, the area under curve (AUC) in 5-year and 10-year survival was 0.74 and 0.72, respectively, in TCGA-BRCA training cohort, demonstrating the survival predicting capability of as-constructed risk model. On the other hand, ROC curves were utilized in validation cohorts. For example, the representative AUC of 5-year and 10-year survival rates were 0.73 and 0.67 in the GSE20685 cohort (Figure 4F), and that in the GSE48390 cohort were 0.81 and 0.72, respectively (Figure 4G). These results suggested that our developed risk model was a dependable prognostic indicator with improved performance. In a nutshell, the combination of the 12-DEGs-related risk signatures in the validation cohorts demonstrated its significant predictive value for prognosis.

### The correlation analysis between DEGs-related risk model and clinicopathological variables

**Figure 5:**
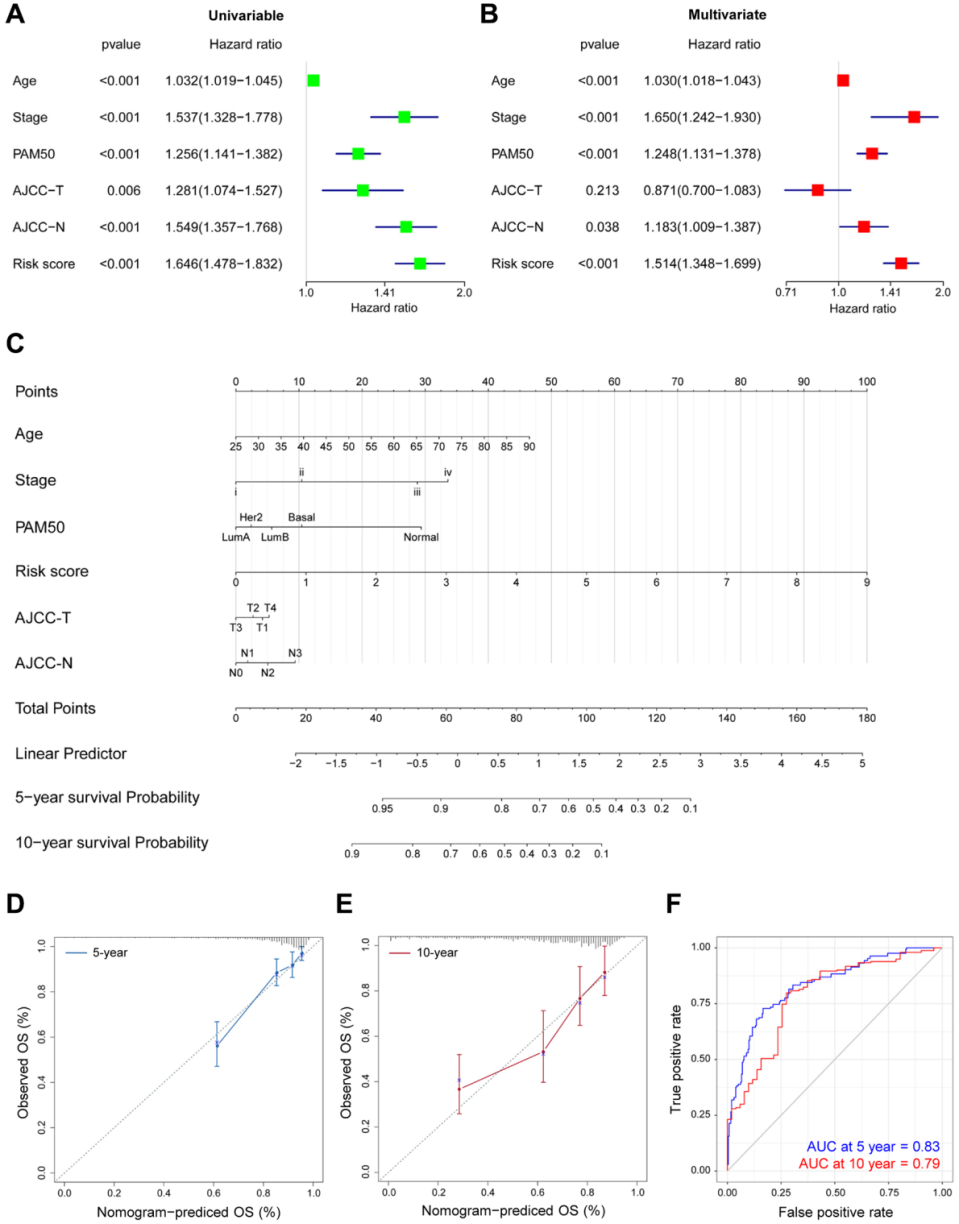
**Prognostic nomogram for predicting survival in TCGA-BRCA patients.** (**A**–**B**) The correlations of the OS risk score and clinical variables by Univariate (**A**) and Multivariate (**B**) Cox regression. (**C**) Prognostic nomogram with certain characteristics in TCGA-BRCA patients. (**D**–**E**) The prediction of 5- (**D**) and 10-year (**E**) survival by calibration curves. x-axis, predicted OS; y-axis, observed OS; the solid line, predicted nomogram; the vertical bars, 95% confidence interval. (**F**) Time-ROC curves for the combination of age, stage, PAM50, T, N and risk score.

Univariate and multivariate Cox regression were subsequently analysed to identify the roles of DEGs-related risk model in predicting prognosis. Univariate Cox regression revealed that there are 6 risk factors for survival prediction, including advanced age, pathological stage, PAM50 molecular subtypes, tumor size, lymph node metastasis, and risk score evaluated by the DEGs-related model (Figure 5A). In addition, multivariate Cox regression analysis demonstrated the high consistence between the OS of BRCA patients and the abovementioned six factors, especially the risk score (Figure 5B). In summary, these Cox regression results demonstrated the functions and contributions of risk score in predicting prognosis without restrictions from tumor clinicopathologic features.

The probability of 5-year and 10-year OS was predicted by generating a nomogram. As shown in Figure 5C, calibration curves demonstrated the favorable consistency in actual and predicted survival performance (Figure 5D–5E), especially for 5-year survival. In addition, after combination of age, pathological stage, PAM50 molecular subtypes, tumor size, lymph node metastasis and our risk score, the predictive accuracy was significantly improved in TCGA-BRCA (Table 1). As evidenced in the time-ROC results in Figure 5F, the comprehensive analysis offered more accurate predictions on the prognosis in BRCA patients (5-year AUC = 0.83, 10-year AUC = 0.79).

**Table 1:** Comparing of the predictive efficiency of the prognostic risk models in the entire training cohorts (*n* = 1076).

**Factor**	**Overall survival**		
	**C-index**	**95% CI**	**AIC**
Age	0.633	0.576–0.690	1640.11
Stage	0.693	0.643–0.734	1628.64
PAM50	0.619	0.567–0.672	1622.68
T	0.608	0.552–0.663	1655.79
N	0.657	0.603–0.711	1634.36
Risk score	0.721	0.670–0.772	1621.76
Age + stage + PAM50 + T + N	0.782	0.737–0.826	1555.89
Risk score + age + stage + PAM50 + T + N	0.811	0.768–0.854	1520.41

Abbreviations: C-index: Harrell's concordance index; CI: confidence interval; AIC: Akaike information criterion.

**Figure 6:**
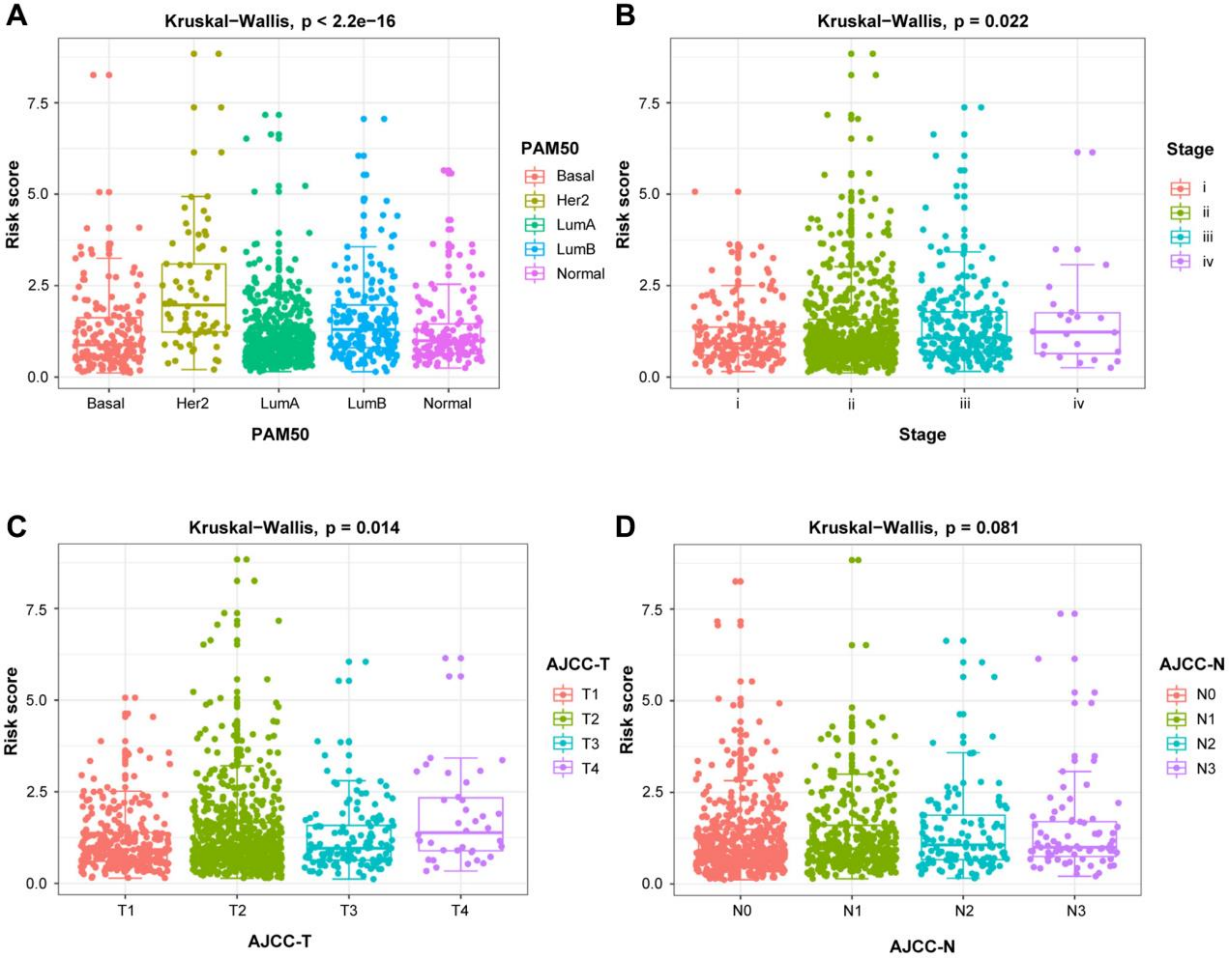
**The relationship between the risk score and clinicopathological variables.** Clinicopathological significance of the prognostic index of BRCA. (**A**) PAM50. (**B**) Stage. (**C**) T stage. (**D**) N stage.

To verify the effect of DEGs-related risk signature for survival on the malignancy of BRCA, our risk model was correlated with clinicopathological variables. In Figure 6, the risk score was significantly increased, when the HER2 subtype and luminal B subtype, the later clinical stage, the larger tumor size, and lymph node metastasis were taken into consideration, confirming the excellent consistency between risk score and prognostic outcomes.

### Exploration of the mechanism in predicted differential risk patients by GSEA

**Figure 7:**
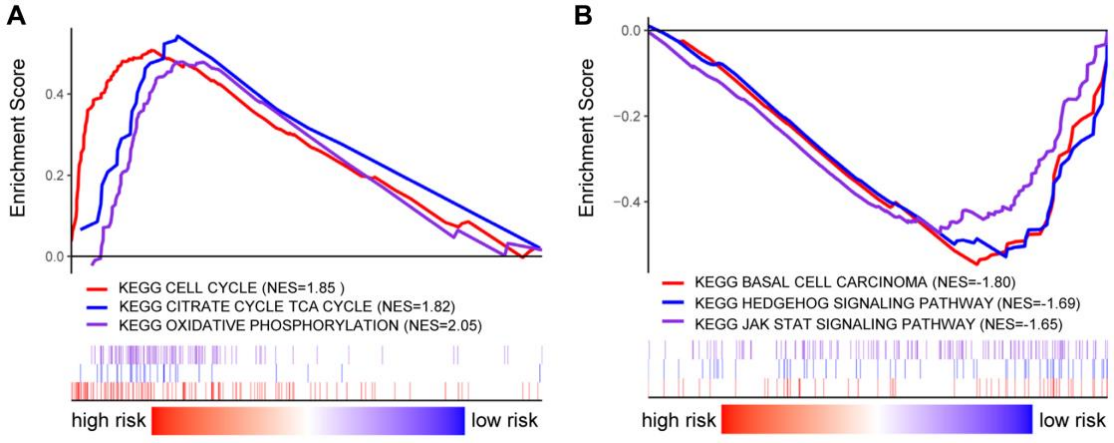
**GSEA analysis in BRCA patients with high- and low-risk score.** (**A**–**B**) GSEA displayed the KEGG enrichment pathways in BRCA patients with high- (**A**) and low-risk score (**B**).

The functional differences among differential risk patients by GSEA were explored by comparing patients in low-risk and high-risk groups. For instance, high-risked patients were positively correlated with cell cycle (NES = 1.85, *p* = 0.012), TCA cycle (NES = 1.82, *p* = 0.016), and oxidative phosphorylation (NES = 2.05, *p* = 0.026). Meanwhile, patients in low-risk groups were negatively correlated with basal cell carcinoma (NES = –1.80, *p* = 0.004), Hedgehog signaling pathway (NES = –1.69, *p* = 0.010), and JAK-STAT signaling pathway (NES = –1.65, *p* = 0.035) (Figure 7). Therefore, the presence of an intensively regulatory role was observed for the development and progression in high-risk BRCA patients, exhibiting significant changes in pathways.

### Prediction of targeted treatment in BRCA patients by our risk score

**Figure 8:**
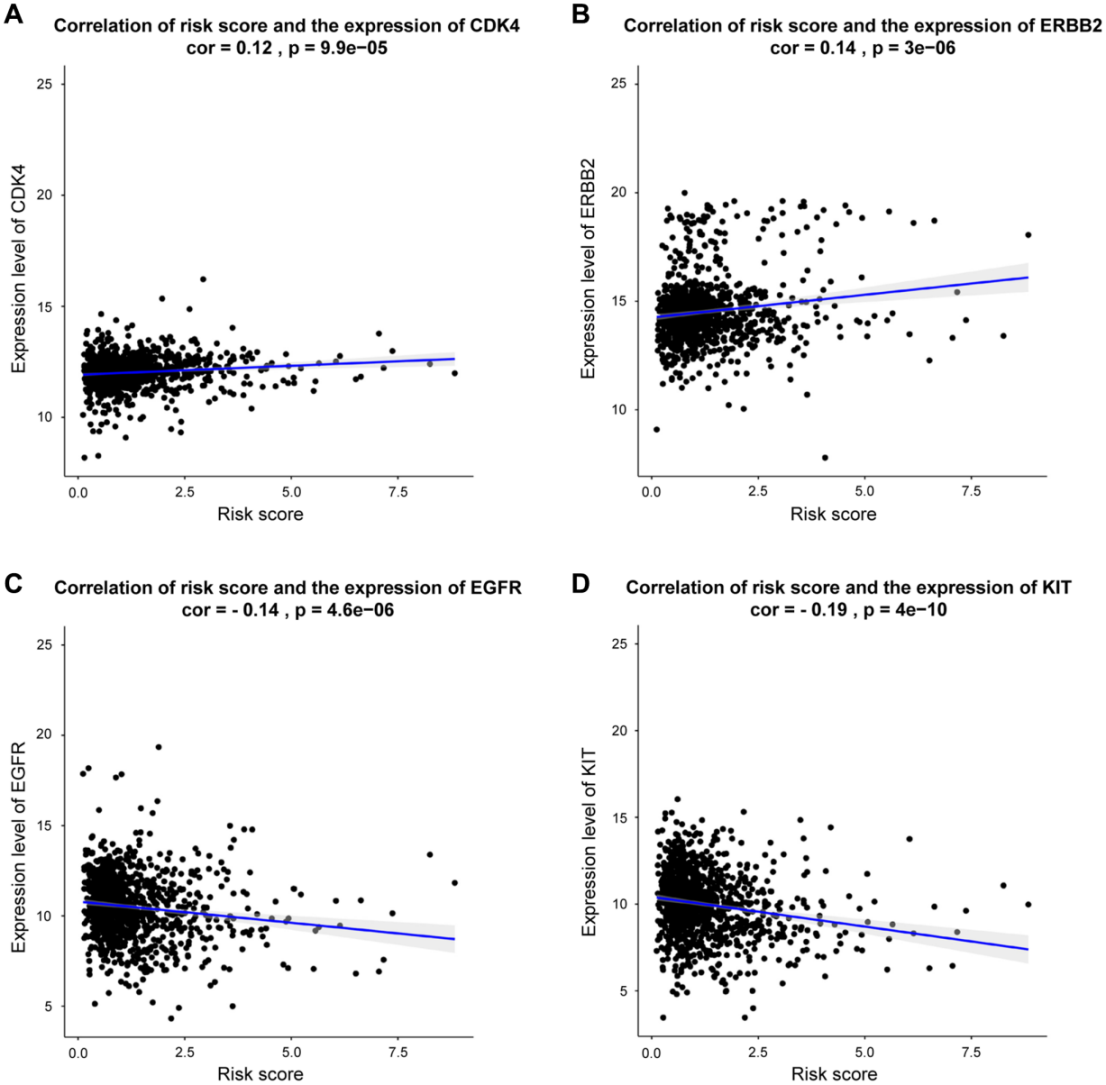
**Correlation between risk score and genes expression for targeted treatment in BRCA.** (**A**–**D**) Correlation analysis shows the results of CDK4, ERBB2, EGFR, KIT, respectively.

As presented in Figure 8, our DEGs-related risk score was closely associated with CDK4 expression (cor = 0.12, *p* = 9.9e-05), as well as the expressions of ERBB2 (cor = 0.14, *p* < 2.2E-16), EGFR (cor = –0.14, *p* <4.6e-06), and KIT (cor = –0.19, *p* < 4e-10) by Pearson’s correlation analysis. In conclusion, patients with higher DEGs-related risk score exhibited favorable therapeutic response to CDK4- and ERBB2- targeted treatments. Otherwise, the lower risk score, patients exhibited better response to EGFR and KIT targeted treatments.

### Verification of the 12-prognostic mRNA expressions between BRCA specimens and adjacent breast tissues by qRT-PCR

**Figure 9:**
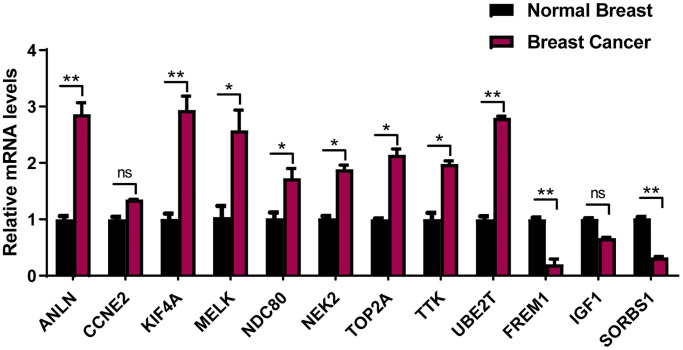
**The expression of the 12 prognostic genes in clinical breast cancer samples.** Clinical samples of breast carcinoma tissues and normal breast tissues were collected and followed by RNA extraction for qRT-PCR measurement of relative gene expression of ANLN, CCNE2, KIF4A, MELK, NDC80, NEK2, TOP2A, TTK, UBE2T FREM1, IGF1 and SORBS1. Data are means ± SEM. ns, denotes not significant. ^*^P < 0.05. ^**^P < 0.001.

To avoid false-positive results from public database, 12-mRNA expressions were further verified based on qRT-PCR results from 20 frozen tissues from BRCA patients (Figure 9). The experimental results indicated that the mRNA expressions of ANLN, KIF4A, MELK, NDC80, NEK2, TOP2A, TTK and UBE2T were upregulated in BRCA tissue in comparison to the adjacent tissues, while that of FREM1 and SORBS1 were downregulated in BRCA tissues compared to the adjacent tissues. These validation results were generally consistent with TCGA-BRCA database (Supplementary Figure 2), suggesting that these prognostic genes played critical roles in the initiative and developing stages of breast cancer.

## DISCUSSION

The aberrant gene expression is a major threat during the progressively developing stages of BRCA, recent intensive studies have indicated that some genes could be potentially targeted for diagnosis, treatment, and prognosis in BRCA. Thus, it is highly desirable to discover effective gene signatures to identify patients' condition, not only to find applicable prognostic targets but also to provide precise therapy for patients at high risk for disease recurrence. Nowadays, full-scale genetic data from BRCA samples can be obtained using DNA microarray and next-generation sequencing, providing comprehensive assessment during diseases progression.

Model in the present study was constructed using five GEO databases and TCGA-BRCA database. Among the total 151 DEGs, 31 DEGs were significantly correlated to the prognosis in BRCA patients, and 12 DEGs (ANLN, CCNE2, KIF4A, MELK, NDC80, NEK2, TOP2A, TTK, UBE2T, FREM1, IGF1 and SORBS1) were recognized and included in the risk model for overall survival prediction. For instance, ANLN was identified as the target for BRCA patients, which correlated with poor survival [11, 12]. CCNE2 promoted G1-S transition in HER2+ BRCA, and hence resulted in trastuzumab resistance [13]. In certain stages of mitosis, KIF4A served as a biomarker for predicting clinical prognosis [14, 15]. MELK promoted the occurrence and progression of colorectal adenocarcinomas [16, 17]. NDC80 was also recognized as a potential target in BRCA [18]. The expression of NEK2 exhibited its importance during the mitotic cell cycle of BRCA [19, 20]. As previously reported, the higher the expression of TOP2A, the better prognosis of BRCA [21]. Also, TTK was another promising therapeutic target due to it was overexpressed in BRCA [22]. UBE2T activated PI3K/Akt signaling pathway and stimulated tumor progression [23]. A recent study proposed that FREM1 isoform was an effective diagnostic and therapeutic marker for BRCA [24]. In certain cancer cells, binding of IGF-1 to IGF-1R could activate some signaling pathway and then promote oncogenic effect [25, 26]. SORBS1 was an adaptor protein involved in cell adhesion, growth factor signaling, and cancer metastasis [27, 28]. Next, our risk model was ascertained to independently predict prognosis in BRCA patients. As the risk score increased, the prognosis outcomes got worsen. The resulting DEGs-related risk model exhibited favorable predictive outcomes in both training (AUC at 5 year = 74%) and validation (AUC at 5 year = 73%) cohorts.

Furthermore, the 12 DEGs-related prognostic model was validated as an effective and excellent indicator of patients' tumor status and prognostic outcomes. Using our risk model, patients with particular clinicopathological features can be stratified into subgroups with varying clinical outcomes. In combination with these results, a nomogram was established by incorporating clinical features and risk score for DEGs signature, which presented excellent performance in survival prediction for BRCA patients. GSEA revealed that inhibited cell cycle is associated with better outcomes, suggesting the critical role of CDK 4/6 inhibitors for the prolonged survival time among BRCA patients. Moreover, the DEGs-related risk score significantly correlated with four targeted therapy genes, providing potential guidance for personalized treatments.

Several prognostic risk models have been previously developed, for example, Zhao et al. recently reported BRCA patients from TCGA cohorts and discussed the immune-related genes and the immune microenvironment of BRCA [5]. Lin et al. identified an autophagy-related genes prognostic model [6]. In our study, a DEGs-related signature was constructed through the different regions and races databases to predict BRCA patients' prognosis, as well as to evaluate the essence in BRCA in a comprehensive manner. The development and progression of BRCA were elaborated from multiple perspectives of differentially expressed genes, and few DEGs have been identified and verified to have potential application in clinics [29, 30]. However, even though external verification was performed in this study, validation of other cohorts is still necessary to verify the prognostic risk model's performance and efficiency. Lastly, the inevitable and inherent bias within the retrospective method should also be addressed.

In conclusion, a DEGs-related risk model was successfully constructed for predicting prognosis in BRCA, which is beneficial for patients and clinical researchers. Our systematic and comprehensive studies suggest that 12-DEGs signature might offer a more accurate evaluation system for BRCA patients' prognosis and provide more personalized therapies. In the future, more extended researches should be carried out to explore the possible mechanisms for the prediction of genes function, as well as the constitutions of the prognostic signature.

## MATERIALS AND METHODS

### Data sources

Firstly, a flow chat was used to illustrate the entire process of our studies (Supplementary Figure 5). A total of 5 GEO datasets were used to identify DEGs in this study. GSE29431, GSE32641, GSE61304, GSE70947, and GSE86374 were downloaded from NCBI-GEO, a free and public database of transcriptional expression. GSE29431 data was obtained with the GPL570 platforms and collected from 54 breast tumors and 12 non-tumor breast tissue samples. GPL887 platforms were utilized to obtain GSE32641 data from 95 breast tumors and 7 normal breast samples. GSE61304 data was obtained with the GPL570 platforms and collected from 58 breast tumors and 4 normal breast tissues. GSE70947 data was obtained with the GPL13607 platforms and collected from 148 breast tumors and 148 normal breast samples. GSE86374 data was obtained with the GPL6422 platforms and collected from 124 breast tumors and 35 normal breast tissues. RNA-seq and survival information of TCGA-BRCA cohorts were retrieved from UCSC Xena [31]. GSE20685 and GSE 48390 data, including 408 BRCA samples from GEO datasets, were used for external validation. Details of the GEO datasets was shown in the Supplementary Table 2.

### Identification of DEGs

Limma, a package that allows users to compare multiple databases in the GEO series under the R environment [32]. DEGs between BC and non-tumor breast tissue samples are identified. Removing the invalid genes, absolute log2 fold change > 1 and adjusted *p* < 0.05 were confirmed as threshold criteria for the genes, to further identify significant DEGs. The volcano plots, Venn diagrams, and heatmap were made by TBtools, and the overlapping DEGs were used to delve deeper [33].

### Functional enrichment analysis of DEGs

Significant DEGs in BRCA were analysed by the “clusterProfiler” package [34], including Gene Ontology (GO) and Kyoto Encyclopedia of Genes and Genomes (KEGG) enrichment analysis [35, 36]. The biological processes, cell components and molecular functions were evaluated. Significant signaling pathways were statistically identified by KEGG analysis, where *p* < 0.05 and adjusted *p* < 0.05 were applied.

### Construction of the PPI network

In this study, STRING online database (version 11.0) was utilized to construct the protein-protein interaction (PPI) network [37]. Cytoscape software (version 3.7.2) was employed to paint the integrated regulatory networks and analyzed the interaction network of different genes [38]. Using the MCODE plug-in component of the Cytoscape software, sorted and filtered critical modules of the whole network [39].

### Construction and validation of OS related prognostic risk model

Univariate Cox regression analysis was firstly carried out to obtain the prognostic DEGs in BRCA, the risk model was subsequently developed using multivariate Cox regression analysis. Final optimization was incorporated into the prognostic risk model via stepwise regression. The resulting risk score was obtained using this formula:

$$\text{Risk score}=\sum_{i=1}^{n}Coef_{i}\times x_{i}$$

where n denotes the number of genes, Coef is the risk coefficient, and x indicates the expression level of individual DEGs in this risk model. In the light of the calculation formula of risk score, the median risk score was critical to divide patients into low-risked or high-risked category. Kaplan-Meier survival analysis was performed to differentiate survival rate between the high-risked and low-risked patients via log-rank test. Followed by the time-ROC analysis, the accurateness of risk model forecast was investigated [40], and verified in the independent BRCA cohorts (GES20685). In the training and validation cohorts, the identical calculation formula was used. Dual Cox regression analysis and clinic correlation analysis were combined to evaluate the effect of risk signature in predicting prognoses of the patients with BRCA.

### Breast cancer gene-expression miner

The expression of 12 selected DEGs in different subtypes of BRCA were analyzed using the bcGenExMiner (version 4.5), by correlating that with the co-expressed genes [41]. The correlation of 12 selected genes was generated using the correlation module.

### Nomogram construction and validation

The “rms” package was used to establish a nomogram in R, which can predict patients’ prognosis by combining the risk score and multiple clinicopathological factors [42]. As for the assessment of predictive accuracy of the model, Calibration curves were established, the concordance index (C-index) and Akaike information criterion (AIC) were further conducted to evaluate the influence of prognosis factors.

### Gene set enrichment analysis (GSEA)

GSEA4.0.3 (https://www.gsea-msigdb.org/gsea/index.jsp) was used to detect which pathways genes are primarily enriched, that is, gene set enrichment analysis differences satisfying the nominal *p* < 0.05 and the FDR < 0.25 were considered statistically significant [43].

### Samples and clinicopathological data

A total of 20 surgically resected breast cancer specimens and adjacent breast tissue were collected from the Second Hospital of Dalian Medical University between January 2010 and January 2018. There are 5 patients involved in each subtypes (luminal A, luminal B, HER2+, and TNBC), identified via pathological examination. No chemotherapeutic or radiotherapeutic treatments have been applied on patients before the surgery. All procedures in this research protocol were approved by the ethics committee in the Second Hospital of Dalian Medical University.

### Isolation of RNA and quantitative reverse transcriptase PCR quantification

Extraction of total RNA and synthesis of complementary DNA was performed by referring to the manufacturers’ instructions. TransStart Tip Green qPCR SuperMix (Transgen Biotech) was utilized for real-time qRT-PCR with specific primers against ANLN, CCNE2, KIF4A, MELK, NDC80, NEK2, TOP2A, TTK, UBE2T FREM1, IGF1, SORBS1, and glyceraldehyde-3-phosphate dehydrogenase (GAPDH), using the ABI 7900HT FAST Real-Time PCR System (Applied Biosystems, USA). GAPDH was selected for normalization. The primer sequences were shown in Supplementary Table 3.

### Statistical analysis

The Wilcox test was proceeded to identify genes’ expression levels of BRCA tissues against that of normal tissues. DEGs in the prognostic risk signature were screened via Cox regression analyses. The log-rank test was performed to correlate OS related Kaplan–Meier survival curve. Time-dependent ROC curve was analysed using the “timeROC” package. Step-comparison for internal-group and external-group was conducted using Mann–Whitney–Wilcoxon test and Kruskal-Wallis test, respectively. The c-index was applied to represent the prognostic nomogram for 5, and 10 years. Statistical significance was considered when two-sided *p*-values were smaller than 0.05, using R software (version 3.6.2).

## Supplementary Materials

Supplementary Figures

Supplementary Tables
