# Supplementary material for: Identification of differentially expressed genes-related prognostic risk model for survival prediction in breast carcinoma patients
Source: Aging (Albany NY). 2021 Jun 26;13(12):16577–99. doi: 10.18632/aging.203178 (PMC8266316; doi:10.18632/aging.203178)
Supplement: Supplementary Figures [file aging-13-203178-s001.pdf]

SUPPLEMENTARY FIGURES

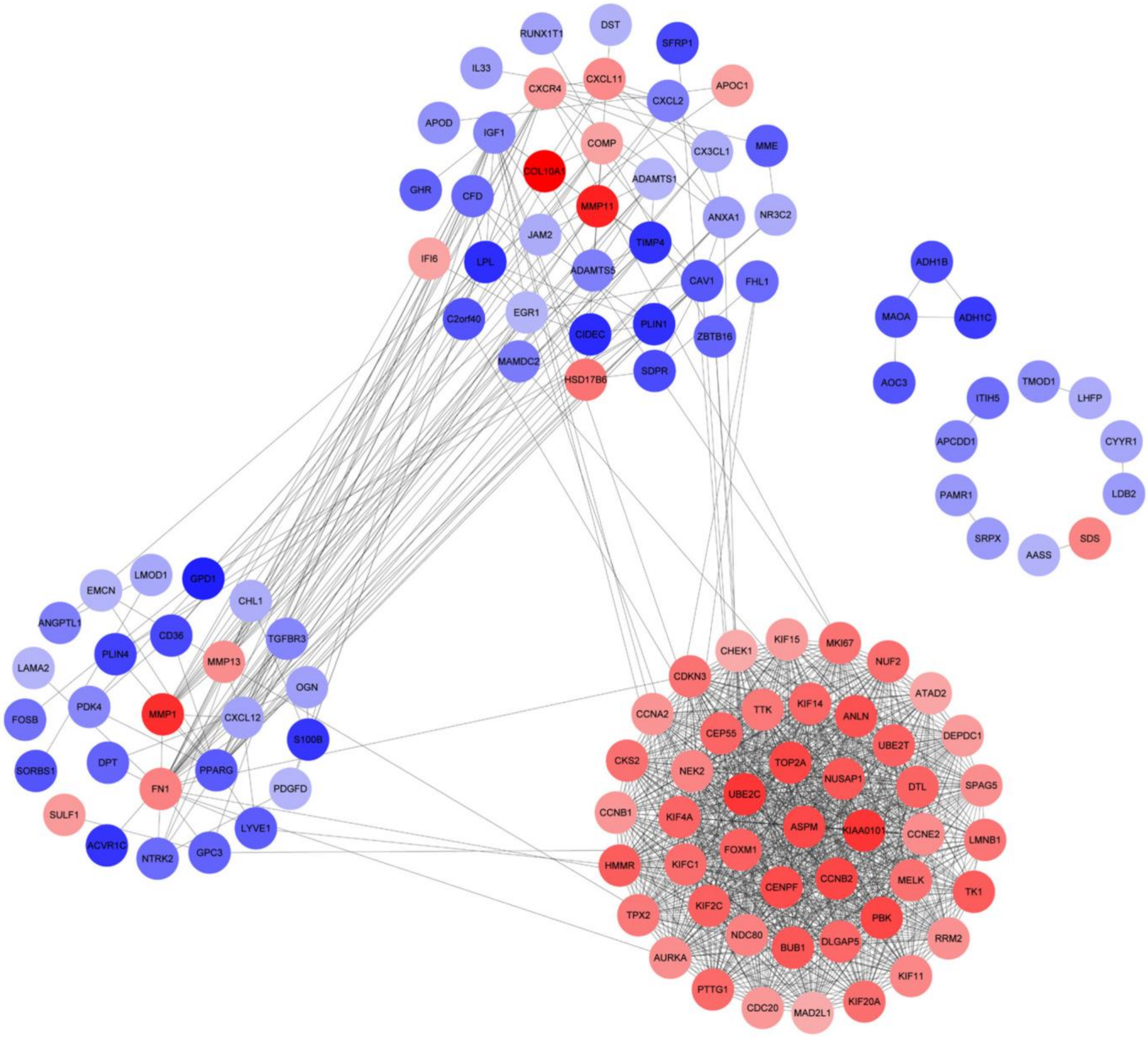

Supplementary Figure 1. PPI network of 151 DEGs was constructed in Cytoscape. Red node indicated the upregulated genes and blue node indicated the downregulated genes. The interaction relationship between nodes was connected by lines.

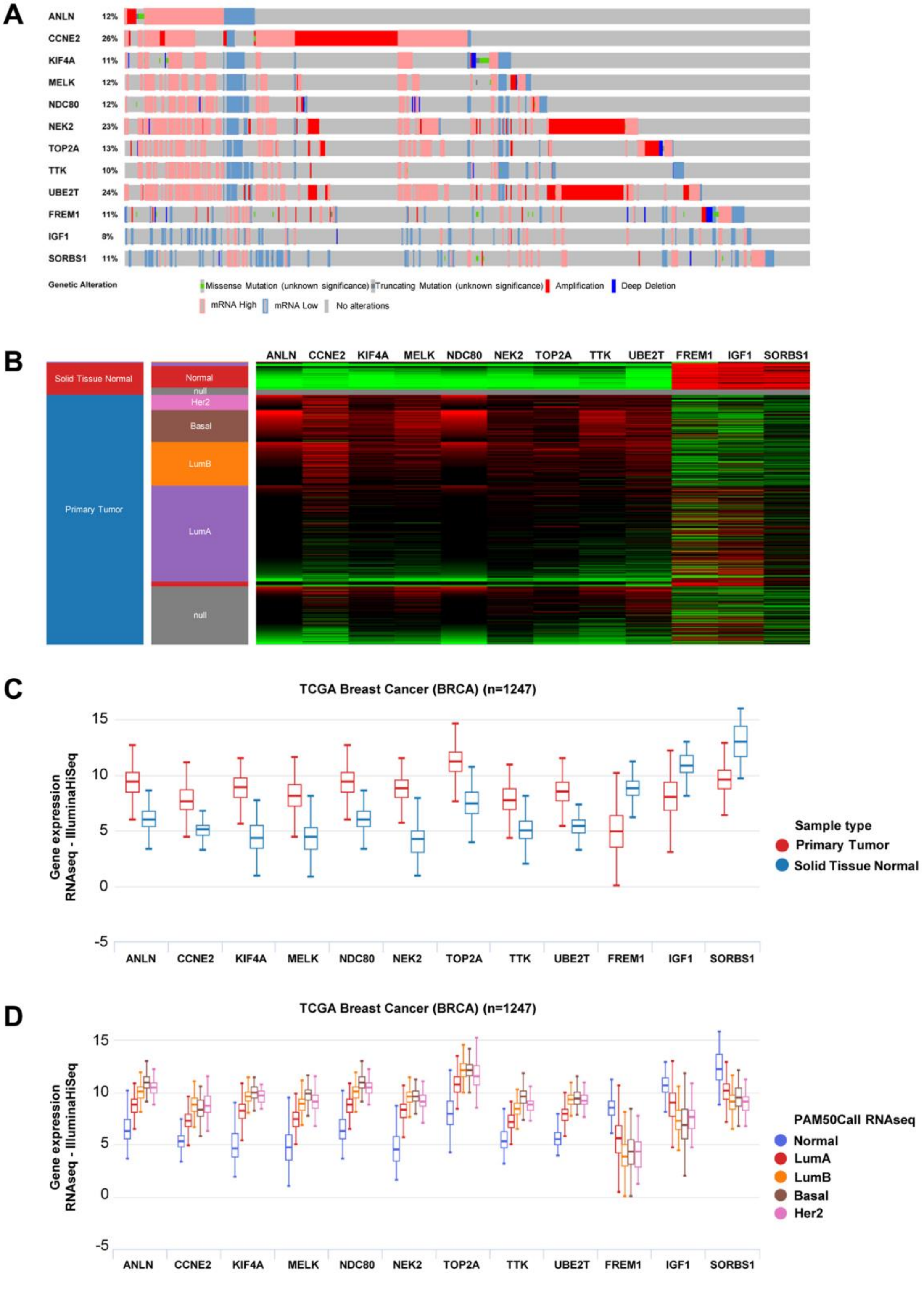

Supplementary Figure 2. Synthetical analyses of 12 selected genes in prognostic risk model. (**A**) OncoPrint demonstrating the copy number variations and mRNA expression alterations of 12 selected genes in prognostic risk model. (**B**) Heatmap of 12 selected genes expression across sample type and PAM50 subtypes in the TCGA-BRCA dataset obtained from the UCSC-Xena online tools. (**C**) Differential expression of the 12 selected genes between normal and BRCA tissues. Red and blue indicate malignant tissues and normal tissues, respectively. (**D**) Differential expression of the 12 selected genes among molecular subtypes of TCGA-BRCA.

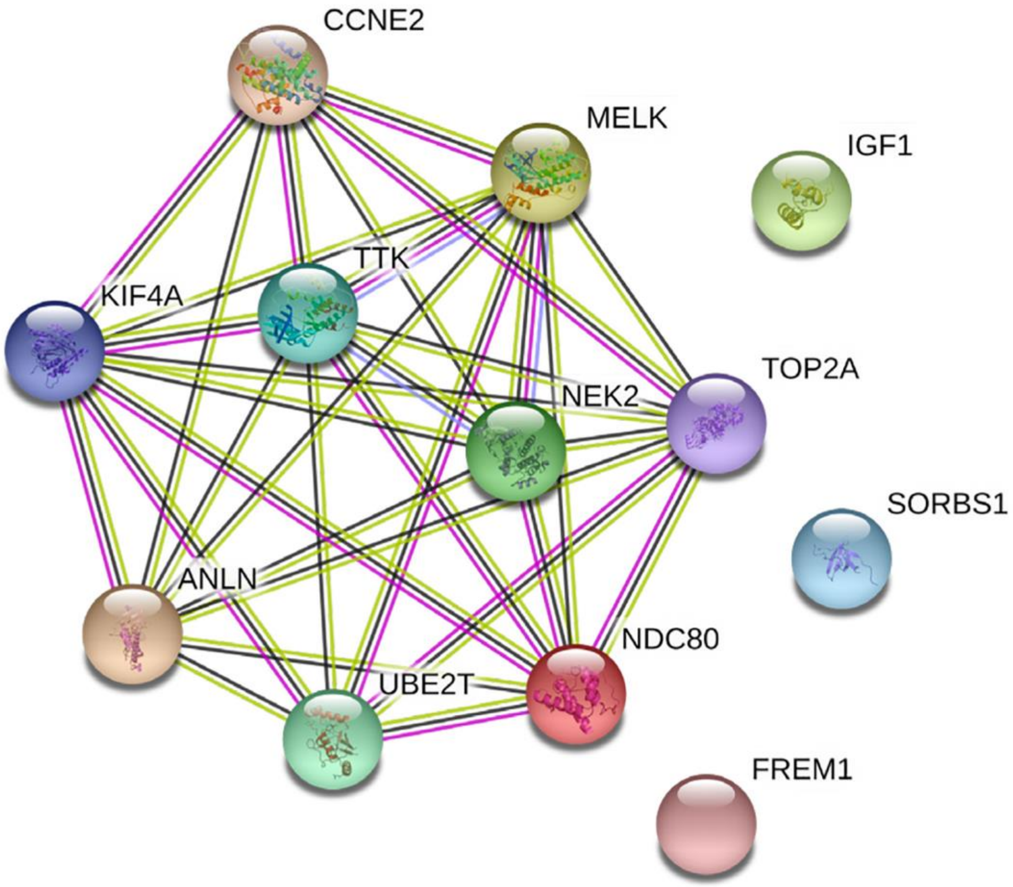

Supplementary Figure 3. PPI analysis of 12 selected DEGs in prognostic risk model by the STRING online dataset.

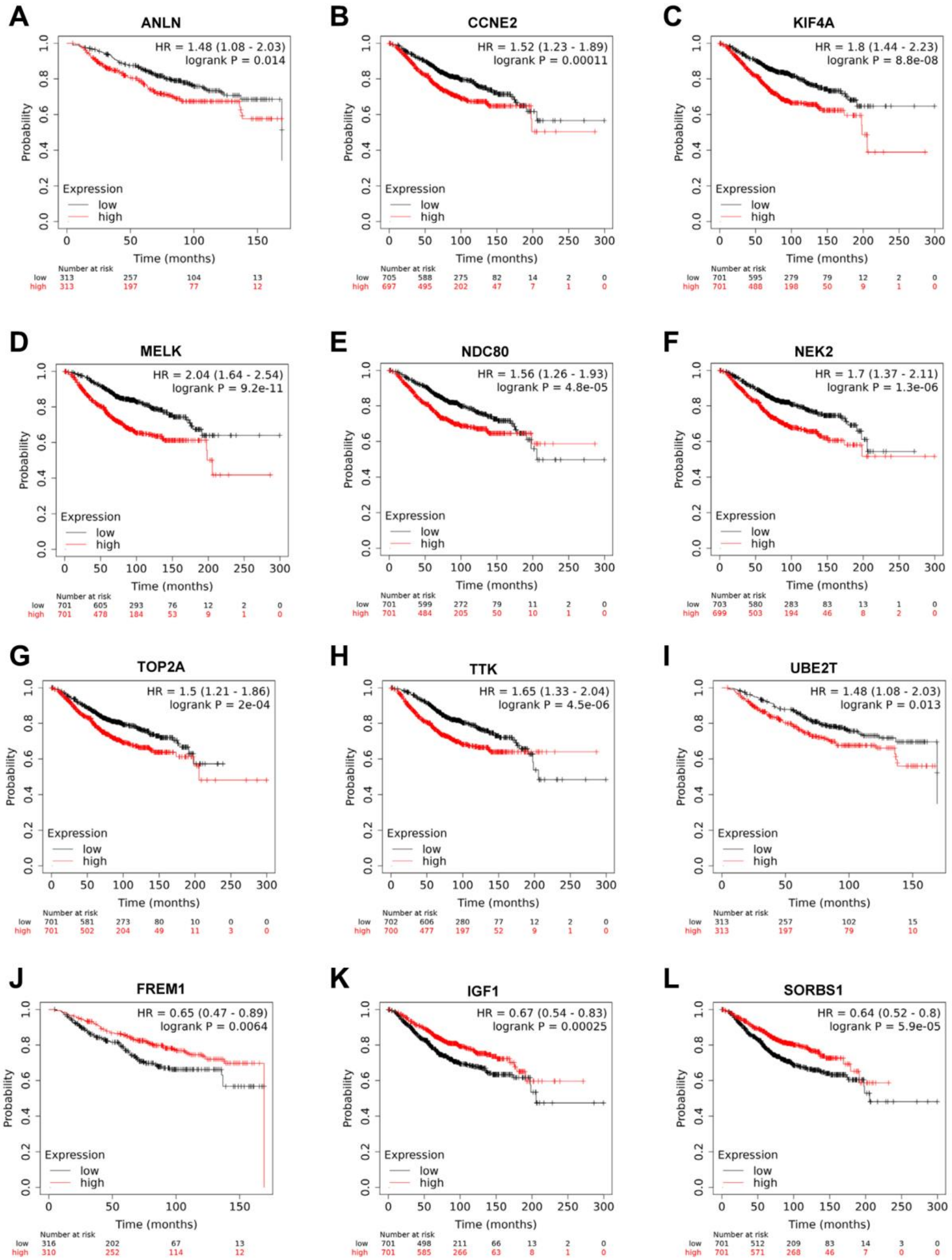

Supplementary Figure 4. Kaplan-Meier survival curves of OS based on 12 genes expression in BRCA patients. (**A**–**I**). Expression level of ANLN, CCNE2, KIF4A, MELK, KNTC2, NEK2, TOP2A, TTK and UBE2T was markedly correlated with poor OS of BRCA patients. (**J**–**L**). Expression level of FREM1, IGF1, SORBS1 was markedly correlated with improved OS of BRCA patients.

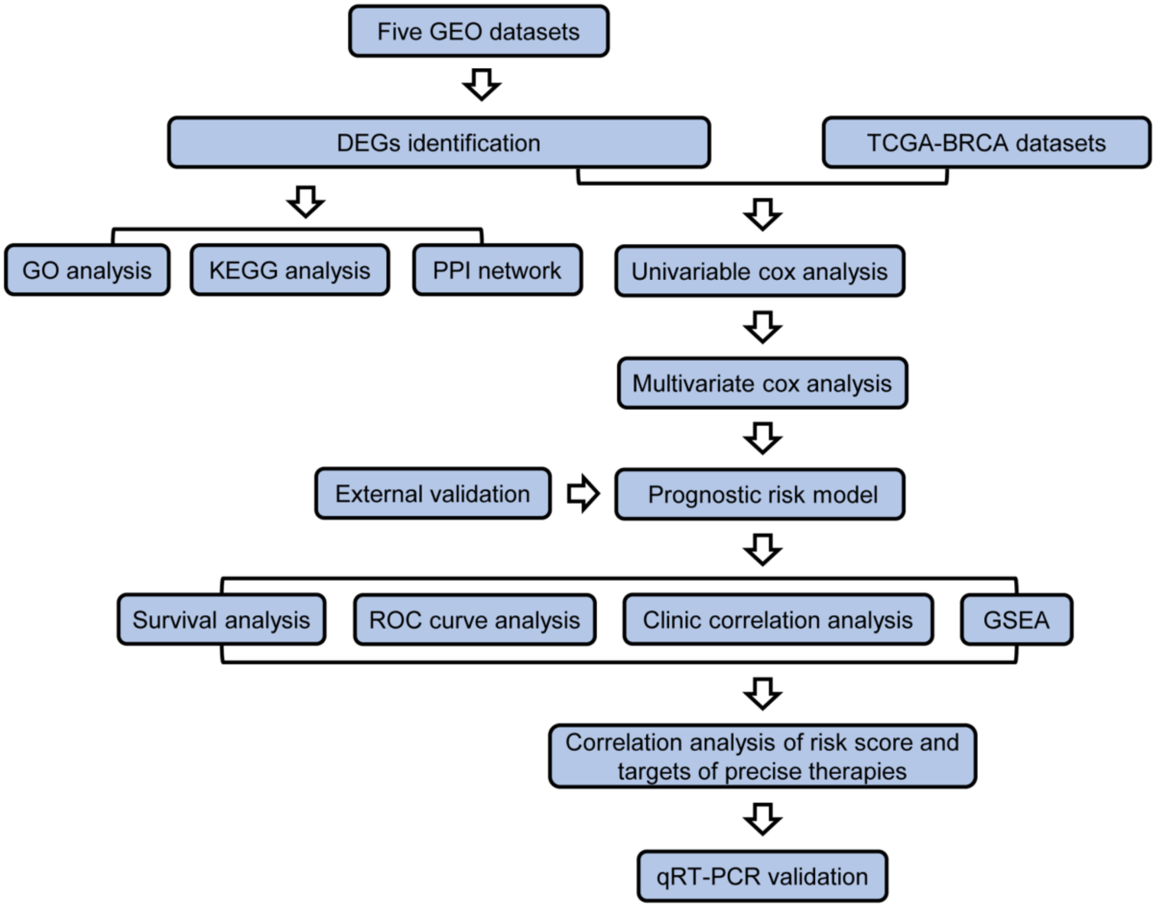

Supplementary Figure 5. The flowchart of data analysis procedures.
