# Supplementary material for: Identification of differentially expressed genes-related prognostic risk model for survival prediction in breast carcinoma patients
Source: Aging (Albany NY). 2021 Jun 26;13(12):16577–99. doi: 10.18632/aging.203178 (PMC8266316; doi:10.18632/aging.203178)
Supplement: Supplementary Tables [file aging-13-203178-s002.pdf]

SUPPLEMENTARY TABLES

Supplementary Table 1. Univariate Cox regression analysis of DEGs.

Gene	Coef	*p*	HR	95%Cl	95%Cl
ANLN	0.422	0.013	1.525	1.093	2.129
CCNE2	0.597	0.001	1.817	1.293	2.554
KIF4A	0.461	0.006	1.585	1.139	2.206
MELK	0.411	0.015	1.508	1.082	2.103
NDC80	0.343	0.042	1.408	1.013	1.959
NEK2	0.425	0.011	1.53	1.101	2.126
TOP2A	0.341	0.043	1.406	1.01	1.958
TTK	0.447	0.008	1.564	1.124	2.176
UBE2T	0.365	0.029	1.441	1.037	2.002
SORBS1	-0.479	0.005	0.619	0.442	0.868
FREM1	-0.357	0.033	0.7	0.504	0.971
IGF1	-0.35	0.04	0.705	0.505	0.984
APOD	-0.347	0.038	0.707	0.509	0.981
LYVE1	0.456	0.008	1.577	1.124	2.212
BUB1	0.375	0.027	1.454	1.043	2.029
CCNA2	0.412	0.016	1.51	1.08	2.11
CCNB2	0.45	0.008	1.569	1.122	2.193
CDC20	0.353	0.036	1.423	1.023	1.978
CEP55	0.367	0.03	1.443	1.037	2.01
FOXM1	0.403	0.017	1.496	1.075	2.084
HSD17B6	0.349	0.036	1.417	1.023	1.964
KIAA0101	0.47	0.008	1.6	1.132	2.262
KIF2C	0.561	0.001	1.752	1.256	2.444
LMNB1	0.419	0.013	1.52	1.092	2.116
MAD2L1	0.374	0.027	1.454	1.043	2.028
MMP13	0.412	0.018	1.509	1.074	2.122
NUF2	0.394	0.019	1.484	1.067	2.063
SQLE	0.403	0.018	1.496	1.071	2.09
TK1	0.341	0.043	1.406	1.01	1.958
TPX2	0.4	0.018	1.492	1.071	2.078
UBE2C	0.469	0.006	1.598	1.146	2.227

Abbreviations: Coef: coefficient; HR: hazard ratio; CI: confidence interval.

Supplementary Table 2. Characteristics of GEO datasets included in the study.

Classification	Series accession ID	Country (region)	Number of samples		Platform ID
			Tumor	Normal	
Identification of DEGs	GSE29431	Spain	54	12	GPL570
	GSE32641	Taiwan	95	7	GPL887
	GSE61304	Singapore	58	4	GPL570
	GSE70947	USA	148	148	GPL13607
	GSE86374	Mexico	124	35	GPL6422
External verification cohorts	GSE20685	Taiwan	327	0	GPL570
	GSE48390	Taiwan	81	0	GPL570

Supplementary Table 3. Primer sequences for qRT-PCR.

Gene Name	Forward Primer	Reverse Primer
ANLN	5′- TGCCAGGCGAGAGAATCTTC -3′	5′- CGCTTAGCATGAGTCATAGACCT -3′
CCNE2	5′- TCAAGACGAAGTAGCCGTTTAC -3′	5′- TGACATCCTGGGTAGTTTTCCTC -3′
KIF4A	5′- TACTGCGGTGGAGCAAGAAG -3′	5′- CATCTGCGCTTGACGGAGAG -3′
MELK	5′- TATTCACCTCGATGATGATTGCG -3′	5′- AGAAAGCCTTAAACGAACTGGTT -3′
NDC80	5′- TCAAGGACCCGAGACCACTTA -3′	5′- GGGAGCTTGTAGAGATTTCATGG -3′
NEK2	5′- TGCTTCGTGAACTGAAACATCC -3′	5′- CCAGAGTCAACTGAGTCATCACT -3′
TOP2A	5′- ACCATTGCAGCCTGTAAATGA -3′	5′- GGGCGGAGCAAAATATGTTCC -3′
TTK	5′- GTGGAGCAGTACCACTAGAAATG -3′	5′- CCCAAGTGAACCGGAAAATGA -3′
UBE2T	5′- ATCCCTCAACATCGCAACTGT -3′	5′- CAGCCTCTGGTAGATTATCAAGC -3′
FREM1	5′- GCCTGTGGTAACCAGGAACAA -3′	5′- CGCAGGTGTATCAGGGTCG -3′
IGF1	5′- GCTCTTCAGTTCGTGTGTGGA -3′	5′- GCCTCCTTAGATCACAGCTCC -3′
SORBS1	5′- ATTCCCAAGCCTTTCCATCAG -3′	5′- TTTTGCTGTTCTCGATTGTGTTG -3′
GAPDH	5′- GGAGCGAGATCCCTCCAAAAT -3′	5′- GGCTGTTGTCATACTTCTCATGG -3′

Abbreviations: qRT-PCR: quantitative real-time polymerase chain reaction.
